# Country v. Town Practice

**Published:** 1890-05-17

**Authors:** 


### The Doctor's Pulpit.

### COUNTRY *V.* TOWN PRACTICE.

Doctors, like farm labourers, have given the country a wide berth during the last dozen or fifteen years. Like the farm labourers also they have discovered that life in large towns is by no means all "beer and skittles." When a live eel is thrown into a frying pan he seldom finds any difficulty in wriggling himself out of it. But when he is out, what better off is he? He has simply transferred himself to the fire. In like manner many a country practitioner, wearied of the long journeys and the longer credit of the country, has invested all his savings in a middle-class practice attached to a corner house in a third-rate street of a fourth-rate suburb of London or other large town. The advantages he pictured to himself before he left Veregreen were many and great. He would become the leading practitioner in his neighbourhood; In course of time he would get attached to a hospital; he would devote his leisure to the study of a specialty; he would never cease until the topmost round of fame's ladder was reached and wealth and honour were his own. His wife and children would have the inestimable advantage of cultivated society; the best schools would be available for both the girls and the boys; his evening leisure would be spent sometimes with men of science, sometimes in the brighter pleasures of the refined social circle. Such and such-like were his dreams.

The picture of the doctor's fancy has been drawn in the simplest of outlines; it can easily be filled in by individual experience. What has the reality proved as compared with the picture? Leisure for the study of a speciality does not fall to the lot of many town practitioners who have young families and no private means. If the ordinary country doctor has never tried a neck and neck race with starvation, he will soon become familiar with such an experience when once he is fairly settled down to a red lamp and a corner house in any general practitioner's part of London. It is not that his income will necessarily be so very small, but that every department of his expenses will be so very great, and his hours of labour so long and unremitting. The scientific men he will be asked to meet, perhaps once in six months, will be the members of the Local Microscopical Society; his evenings out will be few and far between, and the Society will consist mainly of his own patients, one of whom will tell him of Willie's cough, and another will ask him to fix a day for baby's vaccination. Mrs. Robinson and her nerves will be sure to meet him at the drawing-room door, and Mrs. Fidgett and her spasms will certainly sit next to him at supper. The education of the boys and girls he will find to be no such easy matter as he thought. One of his best patients is Mr. Fizzio Graphee, F.R.G.S., who has all the sciences at his fingers' ends. Of course he takes it for granted that the boys will be sent to him, and that in due course the bill for their education will be sent to their trusting parent. What the "education" may consist of it is perhaps best not to inquire. In like manner the Misses Backboard will be delighted to take the girls; and as the Misses Backboard, though never ill themselves, are intimately acquainted with both the churchwardens and all the leading ladies of the congregation, as well as with the rector and the three curates, it would be an act of unparalleled insanity to run the risk of a war with them. Therefore, to the Backboards, and the Backboards only, must the girls go, whatever be the amount of the school fees or the irrelevant and antiquated character of the education.

These are some of the lighter aspects of town disadvantages. We have before, in these pages, dwelt upon other parts of the case; as, for example, the intensely keen competition between medical men in large towns, and the consequent smallness of their fees; the long hours during which the London practitioner must be prepared to suit his patients' convenience; the ill-paid night-work; the abominable stuffiness and unhealthiness of the wretched little living and sleeping rooms of London suburban houses; the impossibility of getting away for a sufficient holiday; the drooping of the wife's health and the flagging of the children's spirits and vitality; all these we have constantly urged before, and we only repeat them now in order that new readers, as well as old ones, may be in possession of the leading facts in our argument.

Nobody has been surprised that country practitioners have migrated in shoals to large towns during the last one or two decades. The country has been in a state of very pronounced chronic poverty. Farmers have not made any money; and as a consequence, landlords have been poor, parsons have been poor, labourers have been poor, and tradesmen have been poor. Two results have flowed from this: all classes have conspired to refrain from calling the doctor in; and when they have called him in they have equally refrained from paying him. Under such circumstances one of two things was inevitable—flight or starvation. The doctor chose flight, and came to diminish the gains, though hardly to lighten the labours of his town rivals.

But everything is changed in the country now. We have it on the authority, not only of statistical returns, but also of experienced agriculturists in Kent, Hertford, Lincolnshire, Yorkshire, Scotland, and other parts of Great Britain, that farming is once more a fairly prosperous occupation. In spite of the diminished value of corn, the prices of all kinds of stock and other agricultural produce are extremely good; labourers are well employed, and fairly paid; landlords receive their rents, and in most counties the parson his tithes; shopkeepers and other tradesmen are moderately prosperous; and the whole aspect of affairs in the country is both bright and promising. There is thus, it is plain, not merely as good a prospect of earning a sufficient income in the country as in the town, but even a better prospect.

Is it necessary to point out to medical men the enormous advantages the country possesses over the town in point of health and pleasure, both for the doctor, his wife, and his children? One is often tempted to ask, whether the average medical man really has any faith whatever in what he so magniloquently calls the "science of hygiene" and the "laws of health"? It is an extraordinary and unaccountable thing that hundreds of fairly respectable practitioners will compete with each other at the East-end of London and in other similar towns until medical fees are cut down to sixpence a consultation, and medicine is charged at less than half the price that a respectable chemist will sell it at, while in many country places the supply of doctors is too small for the needs of the public. If medical practitioners had half as much faith in hygienic science as they profess to have, would not some of them apply that science to their own families, and instead of killing themselves with hard work to make ends meet in large towns, and at the same time poisoning their children with bad air, would they not seek country practices, and live where trees grow, streams run, and the blue sky can be seen? A large, ill-paid practice in a poor town suburb is little better than a living death. Almost any kind of occupation in the country—farming, market gardening, or even the keeping of a chemist's shop, would be infinitely more wholesome, healthier, and happier. When once a man with a family has got into a particular groove no doubt he finds it difficult to get out of it. But any amount of uncertainty and risk in the finding of a better sphere is preferable to the certain poverty and ultimate desperation which await many of the family doctors who practise in the poorer districts of large towns.

